# The lab-confirmed interval of COVID-19 clusters and its application in the strength evaluation of prevention and control measures

**DOI:** 10.1186/s12879-021-05874-6

**Published:** 2021-02-27

**Authors:** Lihong Huang, Liangmin Wei, Yue Jiang, Lijuan Lin

**Affiliations:** 1 Department of Biostatistics, Zhongshan Hospital, Fudan University, 180 Fenglin Road, Xuhui District, Shanghai, 200032 China; 2 Evidence-based Medicine Center, Fudan University, Shanghai, 200032 China; 3 Department of Biostatistics, School of Public Health, Nanjing Medical University, Nanjing, 211166 Jiangsu China

**Keywords:** Novel coronavirus disease, Clustered epidemic, Laboratory capacity, Lab-confirmed interval

## Abstract

**Background:**

The lab-confirmed interval is the date from lab confirmation in a core case (infector) to lab confirmation in a second case (infectee); however, its distribution and application are seldom reported. This study aimed to investigate the lab-confirmed interval and its application in the preliminary evaluation of the strength of disease prevention and control measures.

**Methods:**

Taking European countries and Chinese provinces outside Hubei as examples, we identified 63 infector-infectee pairs from European countries from Wikipedia, and 103 infector-infectee pairs from official public sources in Chinese provinces outside Hubei. The lab-confirmed intervals were obtained through analysis of the collected data and adopting the bootstrap method.

**Results:**

The mean lab-confirmed interval was 2.6 (95% CI: 2.1–3.1) days for Europe and 2.6 (95% CI: 1.9–3.3) days for China outside Hubei, which were shorter than the reported serial intervals. For index patients aged ≥60 years old, the lab-confirmed interval in Europe was slightly longer (mean: 2.9; 95% CI: 2.0–3.6) and obviously longer in China outside Hubei (mean: 3.8; 95% CI: 1.9–5.5) than that for patients aged < 60 years.

**Conclusion:**

Investigation of the lab-confirmed interval can provide additional information on the characteristics of emergent outbreaks and can be a feasible indication to evaluate the strength of prevention and control measures. When the lab-confirmed interval was shorter than the serial interval, it could objectively reflect improvements in laboratory capacity and the surveillance of close contacts.

## Background

In December 2019, a cluster of viral pneumonia cases appeared in Wuhan, the capital city of the Chinese province of Hubei. The outbreak was traced to a novel coronavirus named severe acute respiratory syndrome coronavirus 2 (SARS-CoV-2) [1], and the disease it causes has been named coronavirus disease 2019 (COVID-19). On January 30, 2020, the World Health Organization (WHO) officially declared the COVID-19 outbreak as the sixth public health emergency of international concern [2], following H1N1 (2009) [3], polio (2014) [4], Ebola in West Africa (2014) [5], Zika (2016) [6], and Ebola in the Democratic Republic of Congo (2019) [7]. The outbreak has since spread rapidly to more than 200 countries with an exponentially growing number of confirmed cases. Consequently, the risk assessment at a global level is extremely high [8].

The threat of the unknown virus demanded an adequate response; however, Nirmal Kandel et al. reported that only 57% of 182 countries had the functional capacity to perform crucial activities at the national and subnational levels [9]. In disease prevention, detection, and control, the ability to confirm cases is exceptionally critical. Reliable diagnosis of COVID-19 is based on the detection of unique sequences of viral RNA by nucleic acid amplification tests (NAATs), such as real-time reverse-transcription polymerase chain reaction (rRT-PCR) [10, 11]. Since the publication of in-depth sequencing analysis of SARS-CoV-2 [12], NAAT confirmation testing has been made available. Currently, rRT-PCR-based assays are the most commonly used method for detecting COVID-19 infection. The target genes assayed in various countries/regions are as follows: China (ORF1 ab, N), Germany (RdRP, E, N), Hong Kong (OLRF1b-nsp14, N), Japan (pancoronavirus and multiple targets, S), Thailand (N), France (2 targets in RdRP), and the United States (3 targets in N) [13]. For the evaluation of contacts in COVID-19 cases, PCR detection for asymptomatic or mild contacts may be considered. Therefore, the ability of laboratories to scale-up is crucial in the prevention and control of the epidemic. Moreover, SARS-COV-2 is most likely to be transmitted by very close contacts, such as individuals who share a household, and the WHO has published a protocol for investigating household transmission [14]. Thus, prompt identification of confirmed cases and effective quarantine of those who may have been exposed to the virus are extremely important in stopping the spread of the outbreak.

As the COVID-19 outbreak is still ongoing, evaluation of the strength of prevention and control measures is essential for predicting the progression of the virus globally. The direct indication of laboratory capacity is the PCR detection rate (testing numbers/all potential infected individuals), while the direct indication of isolation efficiency is the isolation rate (quarantine numbers/all potential infected individuals). However, it is difficult to estimate these direct indications accurately due to data limitations and the complexity of the actual situation. An alternative, feasible indication is required for the prevention and control strength evaluation in practice.

Dynamic infectious models, such as compartment models, are a popular method to predict the dynamic progress of an infectious disease. Several dynamic models based on public data have been reported since the initial outbreak of COVID-19 [15–17]. However, obtaining accurate predictions using modelling methods is extremely challenging due to the complexity of prevention and control strategies, virus variation, and difficulty in setting the initial parameters. Wei et al. suggested that dynamic infectious disease models could be applied to evaluate the effect of the prevention and control strategies [18]; however, the modelling method is complex, involving a huge amount of computation, and would be difficult to carry out for most public health workers. Therefore, a more comprehensible and feasible method would be meaningful for the evaluation of the strength of prevention and control strategies during the emergent outbreak.

According to the above reasons, we defined the lab-confirmed interval as the date from lab confirmation in a core case (infector) to lab confirmation in a second case (infectee) in this study. The lab-confirmed interval has rarely been reported, as it may not be a main characteristic parameter of infectious disease. In the present study, taking European countries and Chinese provinces outside Hubei as examples, we investigated the distribution of the lab-confirmed interval to provide additional characteristic information of COVID-19 clusters and applied it as a feasible indication to evaluate the prevention and control strength of the clustered epidemic based on the relationship between the lab-confirmed interval and the reported serial interval.

## Methods

As of December 31, 2020, there were more than 26,235,000 confirmed COVID-19 cases in European countries and around 96,670 confirmed COVID-19 cases in China, which followed the case definition set out in the official diagnostic protocol released by the WHO [11]. To find pairs of infectors and infectees from Europe, we scanned all related websites listed on Wikipedia up to March 30, 2020. Paired records of lab-confirmed COVID-19 cases from Chinese provinces other than Hubei were collected from the publications and websites of national, provincial, and municipal health authorities. All sources of the data are public with open access and no existing conflicts of interest.

We found 63 infector-infectee pairs from European countries and 103 infector-infectee pairs from China outside Hubei province. For each pair, data were extracted and entered into a structured database, including demographic information, date of the first case in the European country/province outside Hubei, and the confirmed dates of the infector and infectee. Each record was extracted and entered by 2 co-authors and was cross-checked to ensure data accuracy.

Continuous variables were described using the means and standard deviations (SD), while absolute and relative frequencies were used to report categorical variables. Lab-confirmed intervals were summarized by days for each investigated area. Additionally, subgroup analyses of the lab-confirmed intervals were also conducted in 2 age groups (index patients < 60 years old or ≥ 60 years old). Since the lab-confirmed interval data included a substantial number of non-positive values, it was assumed to follow a normal distribution, and the 95% confidence intervals for the means and standard deviations were also estimated using the bootstrap method. The trend of the lab-confirmed intervals, along with the duration from the date of the first case in a country/province to the confirmed date of the index patients, was also fitted based on linear regression.

All statistical analyses were performed using R Software Version 3.6.1 (The R Foundation for Statistical Computing).

## Results

The mean age of the index patients was 45.8 years old and 49.2 years old for pairs in Europe and China outside Hubei, respectively. Index patients < 60 years old comprised 57.1% of pairs in Europe and 76.7% of pairs in China outside Hubei. Males accounted for the majority (81.0%) of cases among the European pairs, but the sex distribution for the pairs in China outside Hubei was balanced. (Table 1).

**Table 1:** Characteristics of included patients and lab-confirmed intervals

Characteristics	Europe		China outside Hubei	
	Infector	Infectee	Infector	Infectee
**Pairs**	63		103	
**Age (years)**				
Range	16–87	9–77	20–80	4–90
Mean (SD)	45.8 (19.5)	40.9 (18.2)	49.2 (13.1)	47.5 (19.8)
< 60, n (%)	36 (57.1)	26 (41.3)	79 (76.7)	65 (63.1)
≥ 60, n (%)	14 (22.2)	5 (7.9)	21 (20.4)	26 (25.2)
Missing, n (%)	13 (20.6)	32 (50.8)	3 (2.9)	12 (11.7)
**Sex**				
Male, n (%)	51 (81.0)	12 (19.1)	50 (48.5)	52 (50.5)
Female, n (%)	11 (17.5)	24 (38.1)	50 (48.5)	42 (40.8)
Missing, n (%)	1 (1.5)	27 (42.9)	3 (2.9)	9 (8.7)
**Lab-confirmed interval (days)**				
Mean (SD)	2.6 (2.0)		2.6 (3.9)	
Min, Max	-1, 10		−6, 19	
Median (P25, P75)	2 (1, 4)		2 (0, 4)	
P2.5, P97.5	1, 8		−3, 13	
**Lab-confirmed interval (days) age < 60**				
Mean (SD)	2.4 (1.9)		2.3 (3.8)	
Min, Max	−1, 8		−6, 19	
Median (P25, P75)	2 (1, 3.5)		1 (0, 3)	
P2.5, P97.5	−1, 8		−5, 13	
**Lab-confirmed interval (days) age** ≥ **60**				
Mean (SD)	2.9 (1.5)		3.8 (4.4)	
Min, Max	1, 6		−2, 13	
Median (P25, P75)	3 (2, 4)		2 (1, 5)	
P2.5, P97.5	1, 6		−2, 13	

**Fig. 1:**
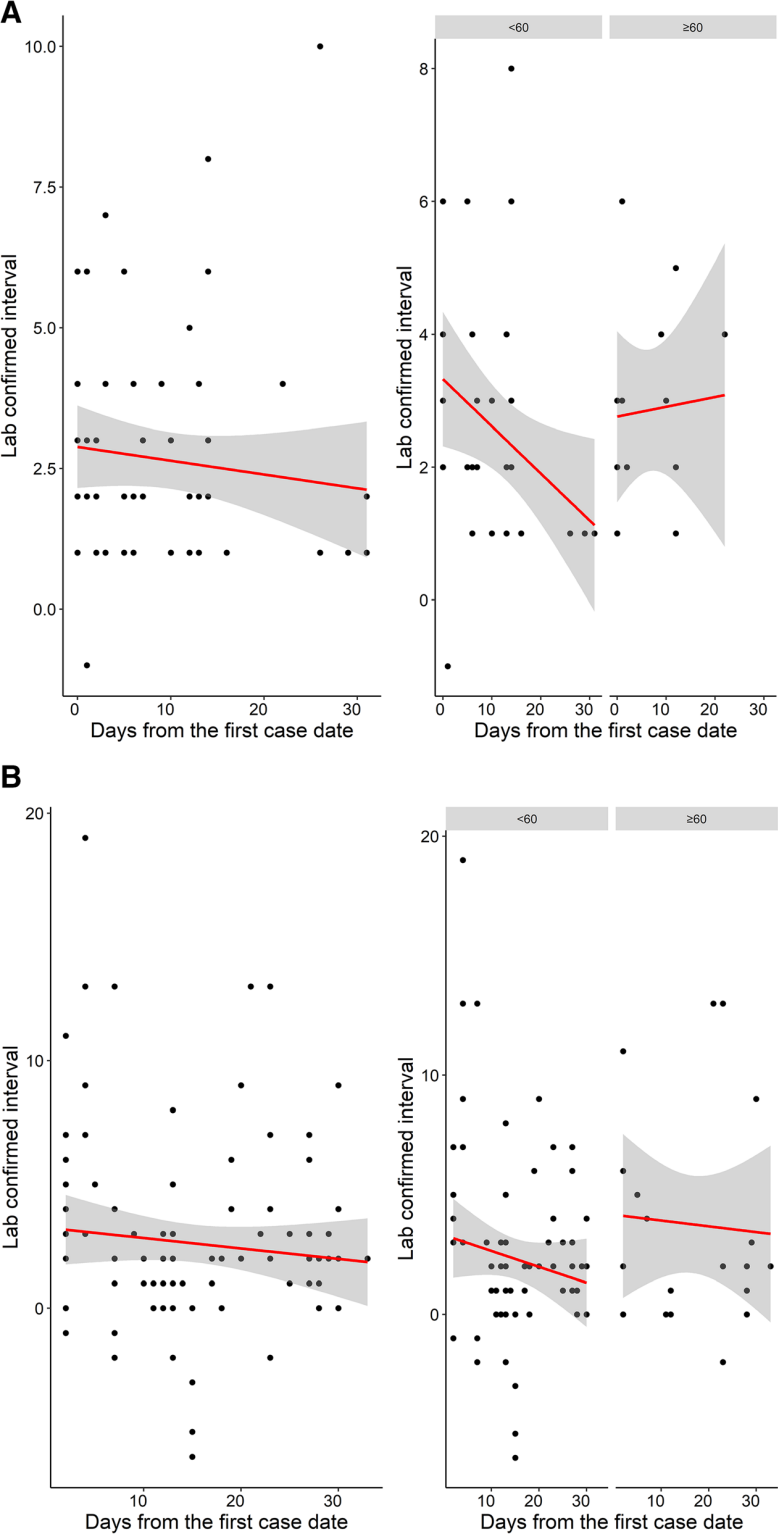
Lab-confirmed interval trend for Europe and China outside Hubei

For the lab-confirmed interval trend, Europe and China outside Hubei were similar, with a slight decrease observed in the first month of the outbreak. Subgroup analysis by age showed obvious decreasing trends in the lab-confirmed interval in the < 60 years group, especially in Europe (Fig. 1).

**Fig. 2:**
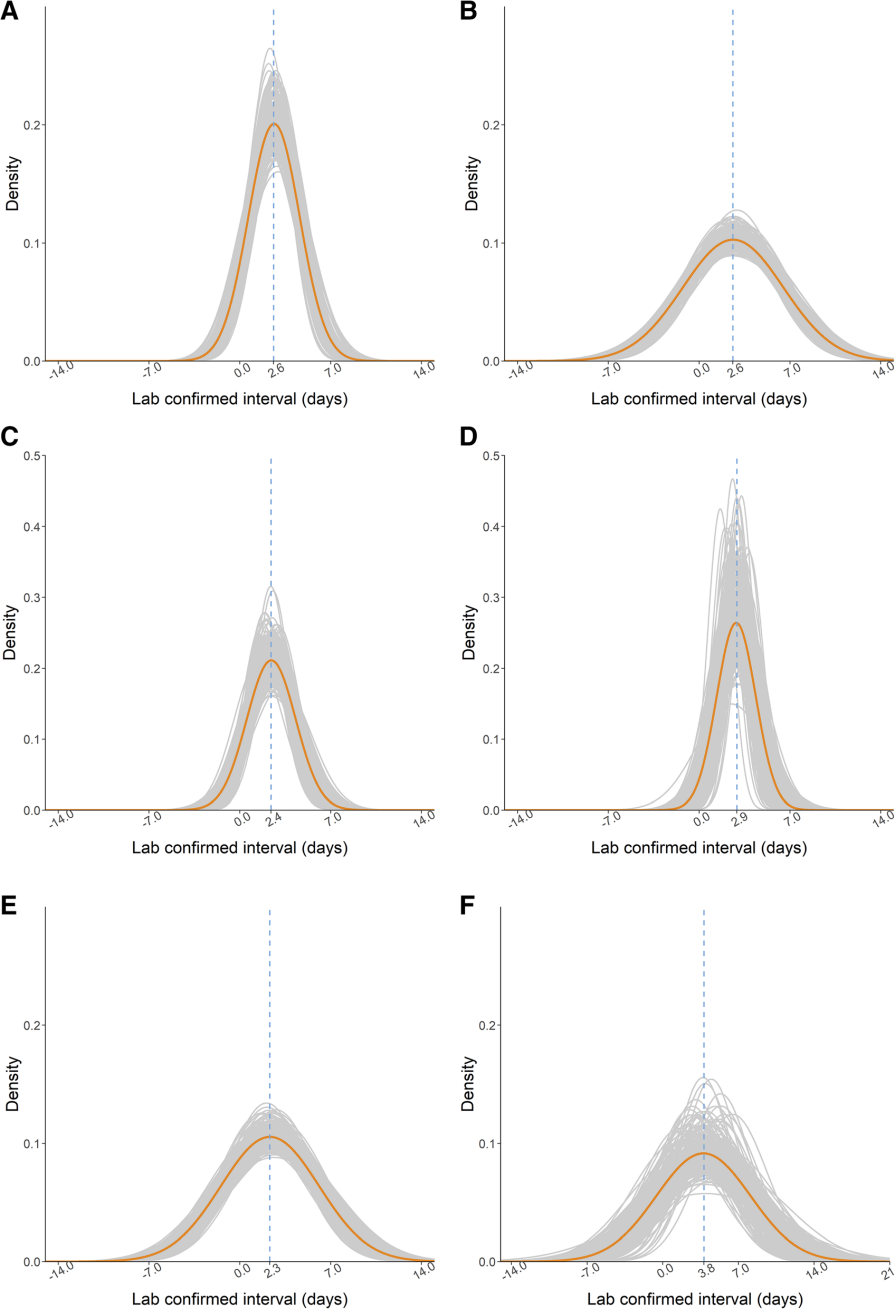
Distribution of lab-confirmed intervals based on bootstrap method

Based on the bootstrap method, we estimated the mean lab-confirmed interval as 2.6 (95% CI: 2.1–3.1) days and the SD of the lab-confirmed interval as 1.7 (95% CI:1.6–2.3) days in Europe. The mean lab-confirmed interval for China outside Hubei (mean: 2.6 days; 95% CI: 1.9–3.3) was similar to that of Europe but showed slightly larger variation (SD: 3.8 days; 95% CI: 3.3–4.3). Interestingly, the lab-confirmed intervals of the index patients < 60 years old were extremely similar in Europe and China outside Hubei. However, in the group of index patients aged ≥60 years old, the lab-confirmed interval was slightly longer in Europe (mean: 2.9; 95% CI: 2.0–3.6) and obviously longer in China outside Hubei (mean: 3.8; 95% CI: 1.9–5.5) than that in the younger group (Fig. 2).

## Discussion

As no specific antiviral therapies are currently available, and efforts to develop antivirals are ongoing, public health measures for managing COVID-19 depend on existing national and regional capacities to prevent, detect, verify, assess, and respond to the outbreak in line with the International Health Regulations [9]. In this study, we defined the lab-confirmed interval as an alternative indicator for evaluating laboratory capacity and cluster outbreak surveillance using Europe and Chinese provinces outside Hubei as examples. In both geographic areas, there was a similar period of time between the first imported cases of COVID-19 and person-to-person community transmission, and the severity of the outbreak depended mostly on the scale of local transmission.

**Fig. 3:**
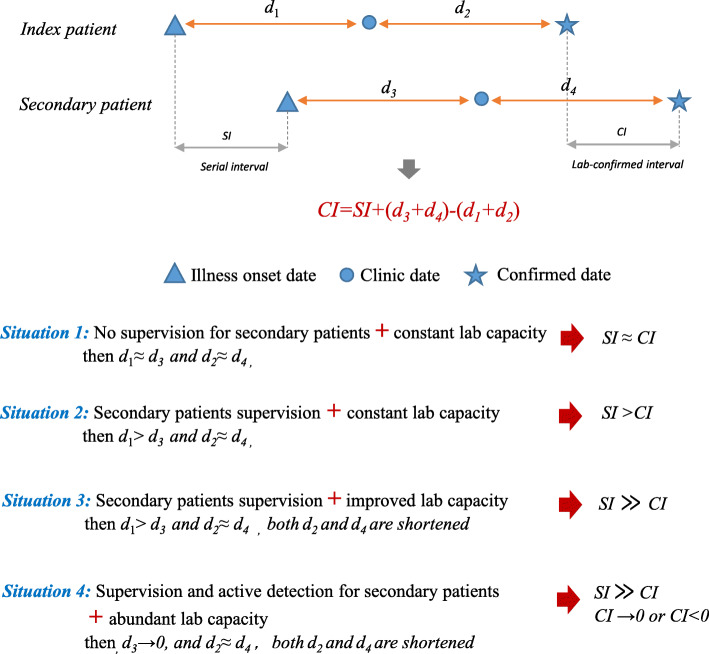
Relationship between serial interval and lab-confirmed interval

The influence of factors other than laboratory capacity on the lab-confirmed interval can easily be questioned. These factors include the date of illness onset and the date of the index patient’s first presentation in clinic, as well as the supervision and scale of laboratory testing of close contacts, and the length of the serial interval (i.e., the time duration between a primary case developing symptoms and a secondary case). However, through comprehensive evaluation of the relationship between the lab-confirmed interval and the serial interval, laboratory capacity and the supervision of secondary patients, which are 2 major aspects in effective prevention and control, can be evaluated objectively (Fig. 3).

Under the premise of the certain serial interval, with a constant laboratory capacity and no supervision of infectees, the lab-confirmed interval should be close to the serial interval (“Situation 1” in Fig. 3). From the results of our study, the estimated lab-confirmed intervals (mean: 2.6 days) were similar in European countries and Chinese provinces outside Hubei, and were shorter than the serial interval estimations of previous studies, which estimated serial interval distributions with a mean of 4.4 days (95% CI: 2.9–6.7) [19], a mean of 3.96 days (95%: CI 3.53–4.39) [20], and a median of 4.0 days (95% CI: 3.1–4.9, [21]). When laboratory capacity was constant (*d*_*2*_ ≈ *d*_*4*_), with sufficient surveillance capability (*d*_*1*_ > *d*_*3*_) of close contacts (suspected infectees), the lab-confirmed interval could be shortened (“Situation 2” in Fig. 3). Meanwhile, with the combination of surveillance capability and improved scale of lab testing, the lab-confirmed interval could be shortened greatly (“Situation 3” in Fig. 3). From the publicly available cluster data we collected from the first month of the outbreak, the estimated lab-confirmed results indicated that the 2 geographic areas investigated were most likely to be “Situation 2” or “Situation 3” (Fig. 3) during that period of the outbreak. A shorter lab-confirmed interval could comprehensively reflect the adequate supervision and timely detection of suspected patients. If the active detection of close contacts (detection before illness onset) can be conducted, the lab-confirmed interval could be close to 0, or even below 0 (“Situation 4” in Fig. 3).

Additionally, we also found that index patients < 60 years old comprised the majority of cases. This could be attributable to younger people engaging in more external activities, such as long-distance business trips or travel, which could increase the risk of infection. Moreover, under the premise of effective isolation and supervision of suspected infectees, our results showed that the lab-confirmed interval of index patients ≥60 years was extended compared to that of young patients. A potential reason for this could be that older people may experience a shorter incubation period or more severe symptoms, and their clinic dates were timely.

In addition to the 2 areas we investigated, there may be other regions in the world with laboratory capacity shortage or with health systems under threat of being overwhelmed by the outbreak [22]. Chinese authorities initially broadened the official definition of SARS-CoV-2 infection to include patients with typical findings on CT. This broader definition has resulted in a higher number of hypothetical cases of COVID-19 and an increasing role for CT in its diagnosis. This approach could offer a reasonable solution for the shortage of laboratory capacity, as the findings of chest CT (e.g., bilateral, basal, and peripheral predominant ground-glass opacity, consolidation, or both) are typical of COVID-19 infection [23].

## Conclusion

Although the sample size of our study was lacking due to the limitations of the publicly available data, our study illustrated that the investigation of the lab-confirmed interval can provide additional information of COVID-19 clusters, such as variation in the characteristics of index patients. When the lab detection rate and isolation efficiency are difficult to estimate directly, under the premise of the certain serial interval, the lab-confirmed interval can serve as an alternative indication of the strength of prevention and control of COVID-19. Through the comprehensive evaluation of its relationship with the serial interval, our study indicated that the estimated mean lab-confirmed interval of 2.6 days, which was shorter than COVID-19 serial intervals reported previously, could objectively reflect the functional capability of a country/province to deal with case confirmation and the management of close contacts. Therefore, we recommend that the lab-confirmed interval is applied as a feasible comprehensive indication to measure the strength of the prevention and control of clustered epidemics preliminarily, as an addition to complex modelling methods. In short, the lab-confirmed interval appears to reflect the strength of prevention and control measures.

## Data Availability

The datasets used and/or analyzed during the current study are available from the corresponding author on reasonable request.

## Abbreviations

CI: Confidence Interval
SARS-CoV-2: Severe Acute Respiratory Syndrome Coronavirus 2
COVID-19: Coronavirus Disease 2019
WHO: World Health Organization
